# *In-vivo* range verification analysis with in-beam PET data for patients treated with proton therapy at CNAO

**DOI:** 10.3389/fonc.2022.929949

**Published:** 2022-09-26

**Authors:** Martina Moglioni, Aafke Christine Kraan, Guido Baroni, Giuseppe Battistoni, Nicola Belcari, Andrea Berti, Pietro Carra, Piergiorgio Cerello, Mario Ciocca, Angelica De Gregorio, Micol De Simoni, Damiano Del Sarto, Marco Donetti, Yunsheng Dong, Alessia Embriaco, Maria Evelina Fantacci, Veronica Ferrero, Elisa Fiorina, Marta Fischetti, Gaia Franciosini, Giuseppe Giraudo, Francesco Laruina, Davide Maestri, Marco Magi, Giuseppe Magro, Etesam Malekzadeh, Michela Marafini, Ilaria Mattei, Enrico Mazzoni, Paolo Mereu, Alfredo Mirandola, Matteo Morrocchi, Silvia Muraro, Ester Orlandi, Vincenzo Patera, Francesco Pennazio, Marco Pullia, Alessandra Retico, Angelo Rivetti, Manuel Dionisio Da Rocha Rolo, Valeria Rosso, Alessio Sarti, Angelo Schiavi, Adalberto Sciubba, Giancarlo Sportelli, Sara Tampellini, Marco Toppi, Giacomo Traini, Antonio Trigilio, Serena Marta Valle, Francesca Valvo, Barbara Vischioni, Viviana Vitolo, Richard Wheadon, Maria Giuseppina Bisogni

**Affiliations:** ^1^ Istituto Nazionale di Fisica Nucleare, Sezione di Pisa, Pisa, Italy; ^2^ Dipartimento di Fisica, Università di Pisa, Pisa, Italy; ^3^ Centro Nazionale di Adroterapia Oncologica, Pavia, Italy; ^4^ Politecnico di Milano, Milano, Italy; ^5^ Istituto Nazionale di Fisica Nucleare, Sezione di Milano, Milano, Italy; ^6^ Istituto di Scienza e Tecnologie dell’Informazione, Consiglio Nazionale delle Ricerche, Pisa, Italy; ^7^ Istituto Nazionale di Fisica Nucleare, Sezione di Torino, Torino, Italy; ^8^ Dipartimento di Fisica, Sapienza Università di Roma, Roma, Italy; ^9^ Istituto Nazionale di Fisica Nucleare, Sezione di Roma, Roma, Italy; ^10^ Dipartimento di Fisica, Università di Milano, Milano, Italy; ^11^ Istituto Nazionale di Fisica Nucleare, Sezione di Pavia, Pavia, Italy; ^12^ Dipartimento di Scienze di Base e Applicate per l’Ingegneria, Sapienza Universit `a di Roma, Roma, Italy; ^13^ Department of Medical Physics, Tarbiat Modares University, Teheran, Iran; ^14^ Museo Storico della Fisica e Centro Studi e Ricerche “E. Fermi”, Roma, Italy; ^15^ Istituto Nazionale di Fisica Nucleare, Sezione dei Laboratori di Frascati, Frascati, Italy

**Keywords:** proton therapy, in-beam PET imaging, *in-vivo* treatment verification, morphological changes, inter-fractional range differences, clinical trial

## Abstract

Morphological changes that may arise through a treatment course are probably one of the most significant sources of range uncertainty in proton therapy. Non-invasive *in-vivo* treatment monitoring is useful to increase treatment quality. The INSIDE in-beam Positron Emission Tomography (PET) scanner performs *in-vivo* range monitoring in proton and carbon therapy treatments at the National Center of Oncological Hadrontherapy (CNAO). It is currently in a clinical trial (ID: NCT03662373) and has acquired in-beam PET data during the treatment of various patients. In this work we analyze the in-beam PET (IB-PET) data of eight patients treated with proton therapy at CNAO. The goal of the analysis is twofold. First, we assess the level of experimental fluctuations in inter-fractional range differences (sensitivity) of the INSIDE PET system by studying patients without morphological changes. Second, we use the obtained results to see whether we can observe anomalously large range variations in patients where morphological changes have occurred. The sensitivity of the INSIDE IB-PET scanner was quantified as the standard deviation of the range difference distributions observed for six patients that did not show morphological changes. Inter-fractional range variations with respect to a reference distribution were estimated using the Most-Likely-Shift (MLS) method. To establish the efficacy of this method, we made a comparison with the Beam’s Eye View (BEV) method. For patients showing no morphological changes in the control CT the average range variation standard deviation was found to be 2.5 mm with the MLS method and 2.3 mm with the BEV method. On the other hand, for patients where some small anatomical changes occurred, we found larger standard deviation values. In these patients we evaluated where anomalous range differences were found and compared them with the CT. We found that the identified regions were mostly in agreement with the morphological changes seen in the CT scan.

## 1 Introduction

Proton therapy is a type of radiation therapy that uses protons to treat cancer. The advantage of proton therapy with respect to conventional radiotherapy (X-rays and electrons) is related to the characteristic depth dose profile of charged particles (Bragg peak) (1). The accuracy of proton therapy strongly depends on the determination of the Bragg peak position. Uncertainties in the knowledge of the proton range can affect the dose distribution. These uncertainties include anatomical changes (physiological or morphological, organ motion, tumour regression, weight loss arising during the course of treatment), patient setup uncertainties, and range errors from uncertainties in CT Hounsfield units (HU), conversion of HU into particle stopping power, and reconstruction artifacts (2–6). In patients where anatomical changes are expected to occur, generally a control CT is acquired at some point during the treatment course (4–9). The scheduling of the control CT is variable and based on the clinical experience of the radiation oncologist. Based on the CT outcome, the radiation oncologist may decide for treatment replanning.

*In-vivo* range monitoring is desirable in order to support the radiation oncologist in the decision on when to perform a control CT (7, 10). One of the most consolidated monitoring techniques is Positron Emission Tomography (PET) (10–13). Nuclear interactions of the therapeutical beam with the tissue result in the production of *β* ^+^ -isotopes, which decay emitting a positron, that annihilates into a 511 keV photon pair. The detection of these photon pairs by means of a PET system yields an activity image, that is indirectly correlated with the dose. Of all PET data acquisition modalities, in-beam (IB) data acquisition is generally considered the most attractive, providing real-time information about the treatment (10, 14, 15).

INSIDE (16) (*INnovative Solution for In-beam Dosimetry in hadronthErapy*) is a bimodal imaging system installed at the National Center of Oncological Hadrontherapy (CNAO), in Pavia, Italy (17). It consists of a particle tracker called *Dose Profiler* (18) and an IB-PET scanner (16). This bimodal architecture allows for the detection of annihilation photons with a PET detector, as well as for the detection of charged particles, also produced as a result of nuclear interactions. Since 2019 INSIDE is under clinical trial. During this trial (ClinicalTrials.gov ID: NCT03662373), we acquired IB-PET data for eight patients that received proton therapy treatments, fractionated in 6 weeks (about 30 sessions) along the entire course of their treatment. The first phase of the trial was completed in March 2020.

The goal of this study is twofold. First, we wish to investigate the level of experimental fluctuations in inter-fractional range differences observed in the INSIDE system, which drives the sensitivity to detect range differences. Such fluctuations can be due to the limited statistics of the acquired PET images, differences in irradiation and data acquisition conditions, PET cart setup errors, fluctuations due to random patient setup errors, and so on. This will be done by studying inter-fractional range shifts for patients that had no morphological changes, using the Most-Likely-Shift (MLS) from Frey et al. (19), where it was applied to offline PET data. To establish the efficacy of the MLS method for IB-PET images, we made a comparison with the Beam’s Eye View method (20). Second, we will compare the results with patients that did show morphological changes, and we will investigate whether it is possible to identify the regions affected by the changes with the MLS method. It must be noted that this is the first study that includes all the available data of the patients treated with proton therapy and monitored with INSIDE. It is also the first time that the MLS method will be applied to IB-PET data.

## 2 Materials and methods

### 2.1 The INSIDE in-beam PET scanner

The design, construction and initial clinical tests of the INSIDE IB-PET system are described in detail in previous works (16, 21–24), and only the most relevant information is given here. The system consists of two planar heads placed at 30 cm from the isocenter, for a total distance between them of 60 cm. Each head has an active area of 10 × 25 cm^2^ and consists of 2 × 5 modules produced by Hamamatsu Photonics (25). Each module is a square matrix of 16 × 16 scintillating crystals of lutetium fine silicate (LFS) (26), each with dimension 3 × 3 × 20 mm^3^. Thus the total number of channels in one PET head is 2560. LFS is a commercial name of the set of Ce-doped silicate scintillation crystals (26, 27). The density (7.35 g/cm^3^) and light yield (80% with respect to NaI:Tl) are comparable to lutetium oxyorthosilicate and LYSO but with improved time performance: it has a ~36-ns decay constant. These crystals are optically coupled one-to-one to silicon photomultipliers (SiPMs). Their temperature was maintained at 18°C with the help of an integrated cooling system.

The 2 × 2560 PET detector channels are read by front-end electronics (FE), based on 64 TOFPET ASICs channels (28), which gives the time stamp of the event encoded through a time-to-digital converter with a resolution of 50 ps. The energy information was obtained with the time over threshold (TOT) method. The signals from the FE ASICs are processed locally by Field Programmable Gate Array (FPGA) boards. The channel dead time is imposed by the FE and is below 300 ns for a 511 keV event (28). The energy resolution for 511 keV photons was ~13%, as measured with a *β* ^+^ source of ^22^Na. The measured coincidence time resolution (sigma) was 450 ps. The acquisition is performed all along the treatment during both irradiation (spill) and beam pauses (inter-spill). However, in this analysis we have only used the data acquired during the inter-spill beam pauses, as well as data acquired a short time after the delivery of the field (see Section *Pre-processing*).

**Figure 1:**
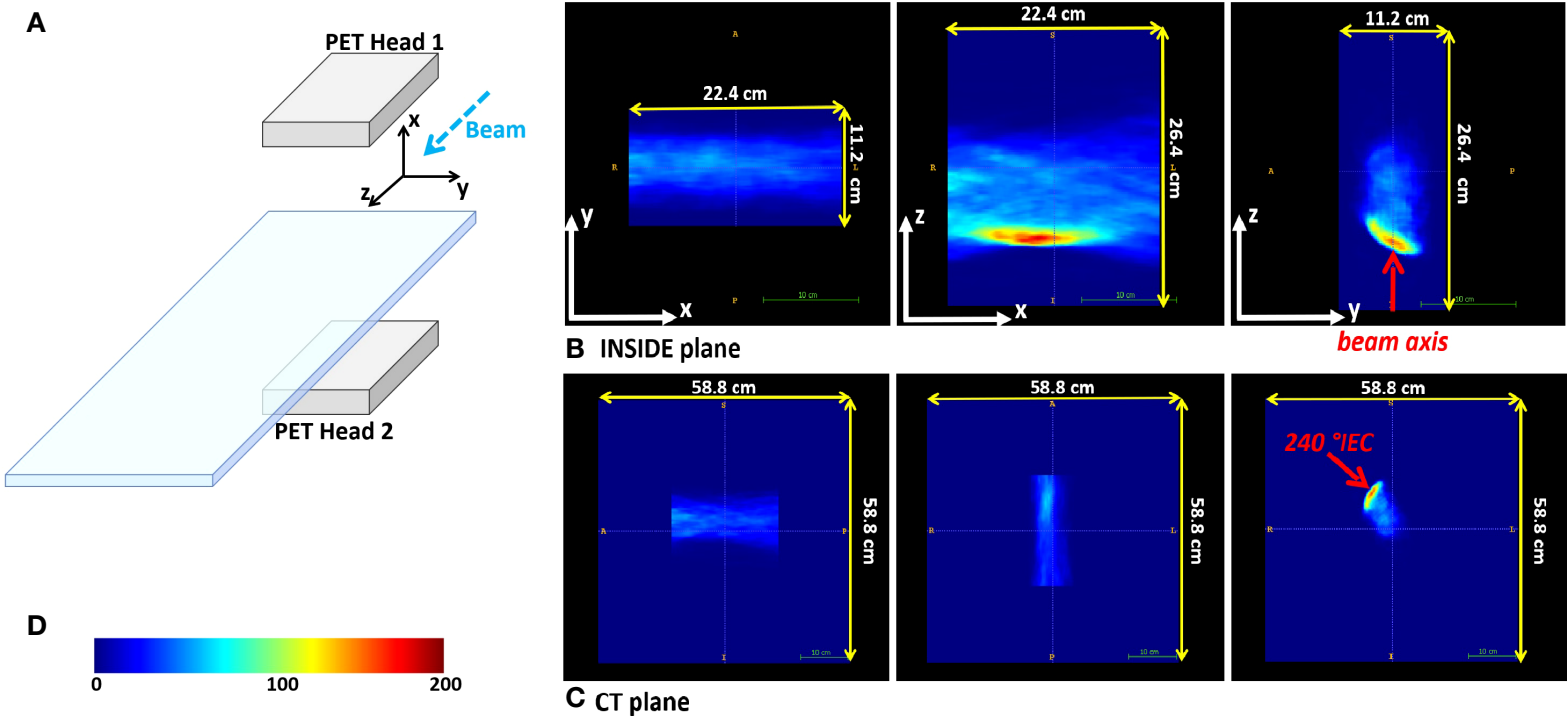
Reference frames. **(A)** Sketch of the INSIDE setup and reference frame. **(B)** Example of the three orthogonal projections of a reconstructed PET image for patient 002P (see 2.3). **(C)** The same reconstructed PET image in the CT reference frame. In **(B, C)** the beam axis is indicated with the red arrow. **(D)** The color-scale of the PET images.

The 3D image reconstruction is performed on-the-fly with an iterative multicore Maximum Likelihood Expectation Maximization (MLEM) (29) algorithm, and its results are provided online during the treatment. Advanced analysis of the PET images is done offline (see Section *Analysis*). The reconstructed image FOV is 224 × 112 × 264 mm^3^, with a voxel size of 1.6 × 1.6 × 1.6 mm^3^. The IB-PET reference system is reported in **Figure 1A**, where the *z* axis is parallel to the beam direction. **Figure 1B** shows a PET image in the three main projections. Due to the partial angular coverage of the PET system, the images suffer from artefacts in the direction perpendicular to the planes (30), as well as from limited statistics [see also (31)].

### 2.2 Dose delivery at CNAO

At CNAO protons are delivered with a modulated pencil beam scanning technique in a fixed beam-line, while the patient couch can rotate. The Dose Delivery System (DDS) (32) guides and monitors the proton beams accelerated by the synchrotron, and distributes the dose with a full 3D scanning technique. The dose results from the superposition of a large number of beams conveyed to the tumour volume. The irradiated volume is divided into several energy layers (slices), each of which is irradiated by various overlapping iso-energetic beams (spots) arranged in a grid of 3 mm pitch. A typical proton therapy treatment has 20 to 30 fractions. Each treatment fraction is delivered through two or more fields, each consisting of many thousands of spots to cover the target. For more details about the beam specifications, scanning technique and the CNAO particle accelerator we refer to previously published works (17, 32).

The DDS provides a log-file in which the information about the actually delivered particles is stored, including the number of delivered primaries, energy and the lateral position of the spot. The information from this log-file was converted into a binary mask in the same reference frame and with the same voxel size as the PET images (see **Figures 1A**, **B**), in which the non-zero values define a volume-of-interest, where PET activity can be present. This volume-of-interest will be referred to as the expected activity mask. The dose delivery time for the patients was typically a few minutes. The temporal structure of the beam extraction was 1 s of spill, during which the protons are delivered, and 2 s of pause between spills (inter-spill periods). The INSIDE PET system is mounted on a cart that is positioned before data acquisition. The position accuracy is about 1 mm, thanks to a dedicated cart positioning system. The presence of the cart has limited the number of patients that could be enrolled in the trial: it was only possible to monitor patients with beam angles without mechanical incompatibilities of the INSIDE setup with the patient couch movements.

### 2.3 Patient dataset

We analyzed the IB-PET data of the patients treated with protons. A patient treated with protons at the CNAO typically receives a total dose of 60 Gy divided into fractions of 2 Gy each.

**Table 1** reports the patients analyzed, with the patient ID, the pathology, the number of fields *N_fields_*, the number of treatment fractions delivered *N_frac_*, the number of treatment fractions with PET data that could be included in the analysis, *N_PET_*, the control CT outcome, as evaluated by the radiation oncologist, and the monitored treatment angle. The control CT is acquired approximately during the third week from the start of treatment. Two of the monitored patients (006P and 007P) showed morphological changes, nevertheless, they were not replanned because the treatment plan quality was still deemed acceptable. In this work we used the PET data for each patient in **Table 1**, that were acquired during the first field delivered. In this way contamination of the activity from the other fields was avoided. The choice of the field was made by clinical personnel.

**Table 1:** Patients treated with protons analyzed in this work, with patient ID, pathology, number of fields, number of fractions delivered, number of fractions monitored, the control CT outcome, as evaluated by the radiation oncologist, and the monitored treatment angle.

Patient	Pathology	*N_fields_*	*N_frac_*	*N_PET_*	Control CT	Monitored
ID					outcome	angle [°IEC]
002P	Meningioma	2	30	12	unchanged	240
003P	Meningioma	2	27	9	unchanged	235
004P	Meningioma	2	31	8	unchanged	245
005P	Chordoma	2	27	10	unchanged	5
006P	Adenoid Cystic Carcinoma	2	35	10	changed	175
007P	Adenoid Cystic Carcinoma	3	33	5	changed	270
008P	Adenoid Cystic Carcinoma	3	33	11	unchanged	0
009P	Chondrosarcoma	3	27	10	unchanged	0

**Figure 2:**
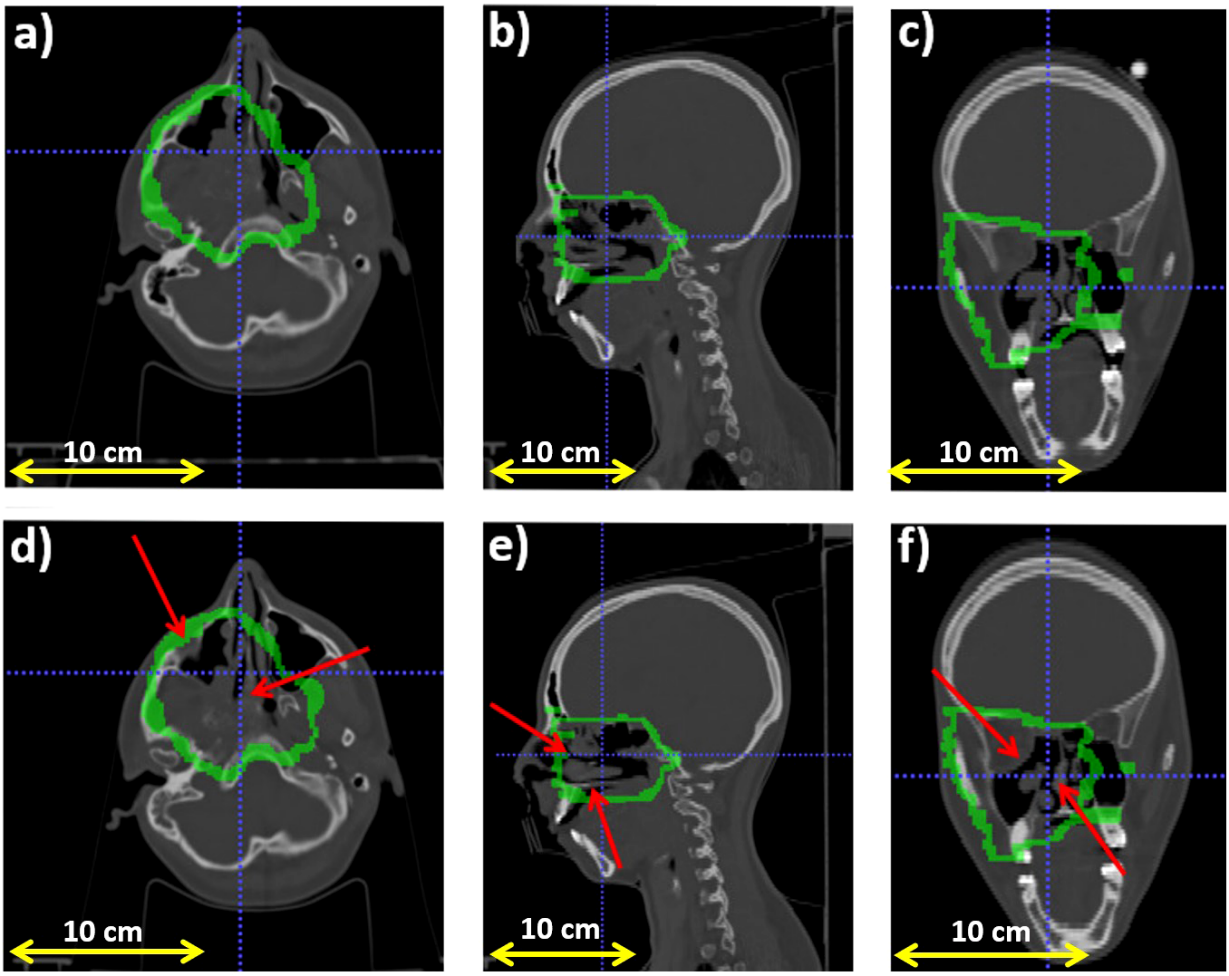
Planning CT (upper row) and control CT (lower row) for patient 006P. **(A)** Axial, **(B)** sagittal and **(C)** coronal views for the planning CT, and **(D)** Axial, **(E)** sagittal and **(F)** coronal views for the control CT. The morphological change is highlighted with a red arrow. Also, the CTV (Clinical Target Volume) area is highlighted with a green line.

**Figure 3:**
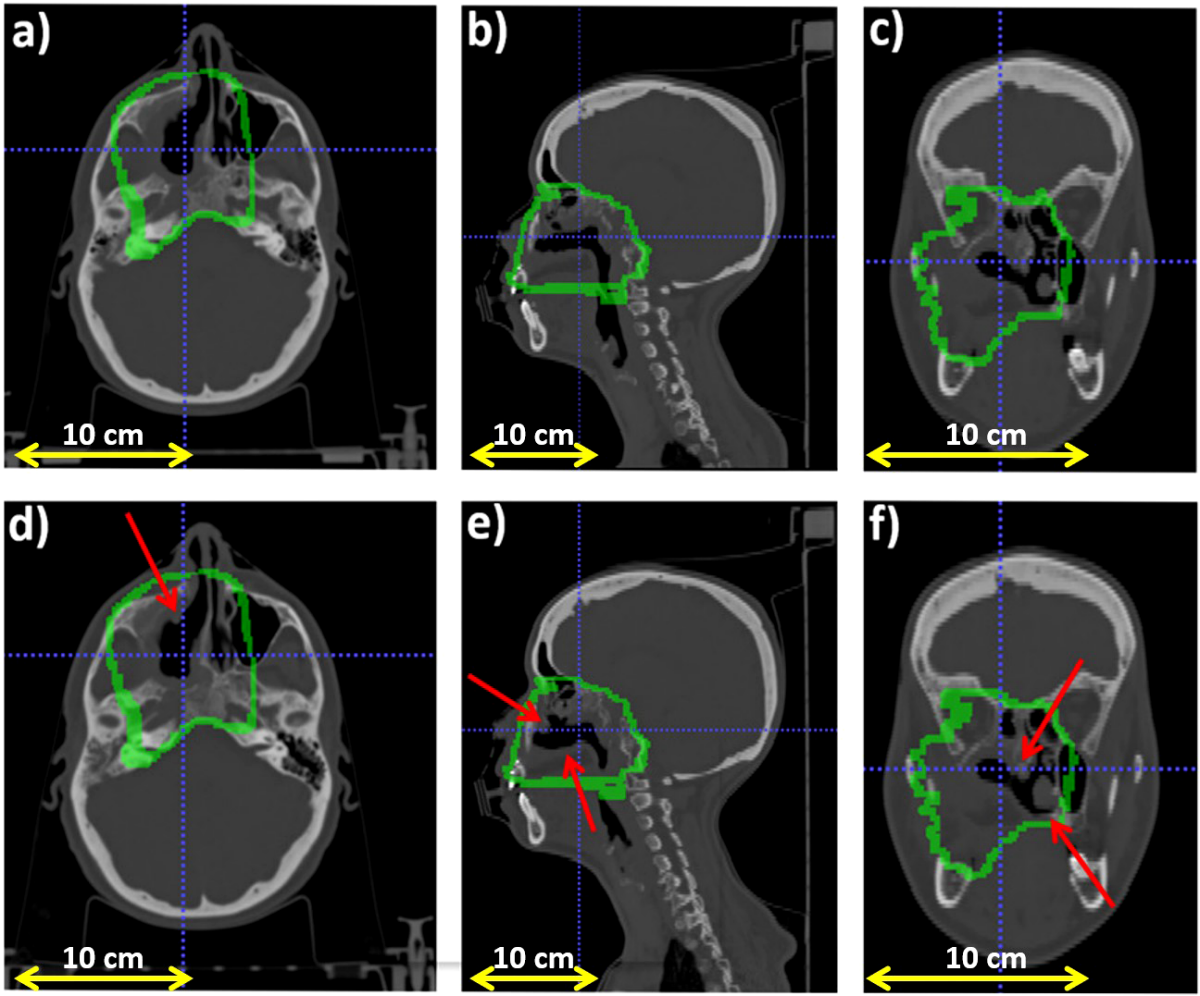
Planning CT (upper row) and control CT (lower row) for patient 007P. **(A)** Axial, **(B)** sagittal and **(C)** coronal views for the planning CT, and **(D)** Axial, **(E)** sagittal and **(F)** coronal views for the control CT. The morphological change is highlighted with a red arrows and the CTV (Clinical Target Volume) area is highlighted with a green line.

**Figure 2** reports the planning CT scan (upper row) and the control CT (lower row) of patient 006P. **Figure 3** reports the same for patient 007P. In this case there are two anatomical modifications: a mild emptying in the GTV for a possible tumor response and also some inflammation reaction (mucositis) in the CTV within the nasal cavity.

### 2.4 Pre-processing

**Figure 4:**
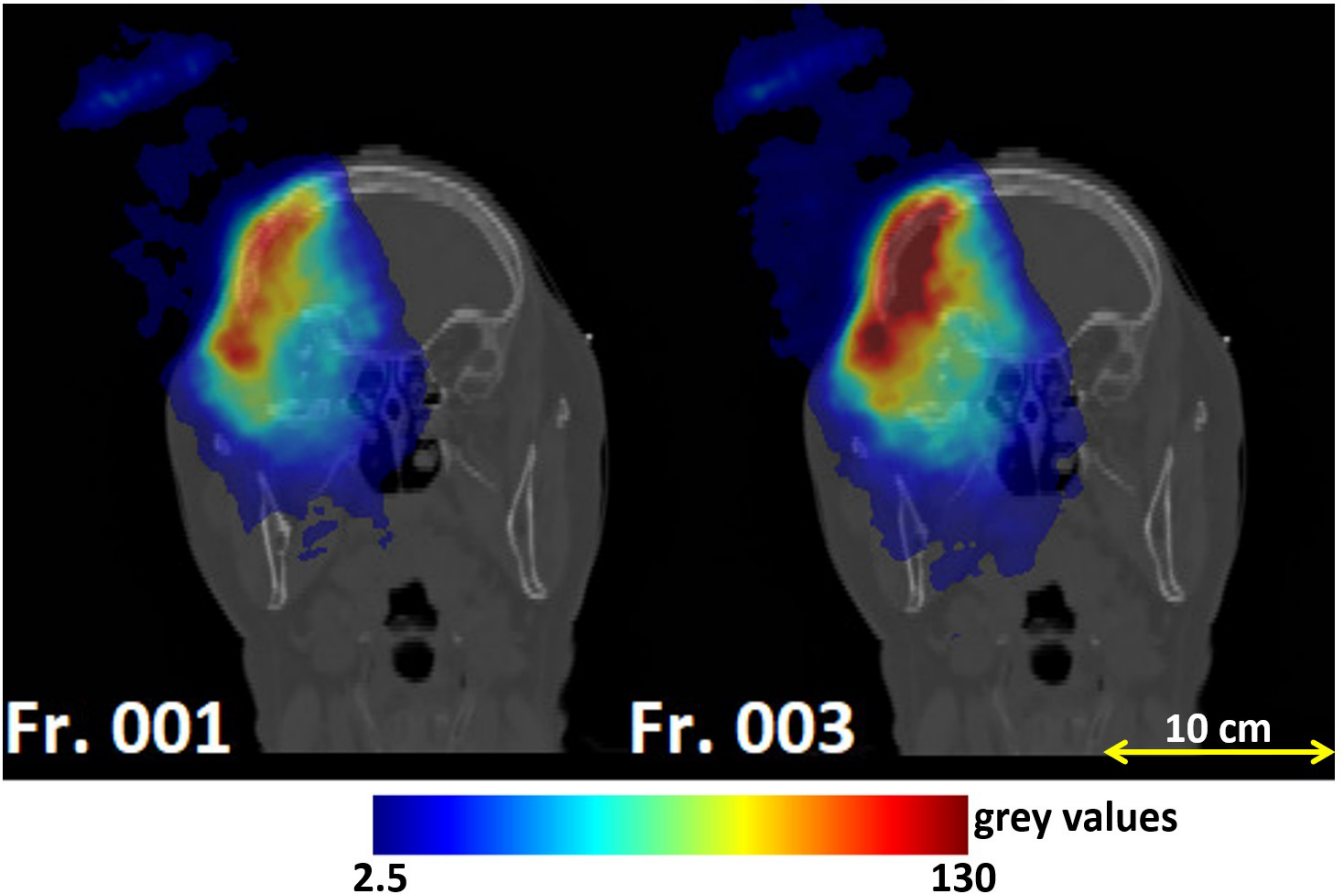
Coronal views of the planning CT of patient 004P: reconstructed PET images corresponding to the monitored fractions 1 and 3. The left and right image had different dose delivery and acquisition times, resulting in different activities. Fraction 3 had to be excluded because of the larger activity compared to the other fractions.

In this work we used the IB-PET data acquired over the time-interval from the start of irradiation until 10 seconds after the end of the irradiation, whereby during irradiation only the inter-spill data were used. Although at the CNAO synchrotron the exact number of spills used for a given treatment is quite stable, small variations are possible from day-to-day, depending on the accelerator conditions. Therefore the reconstructed activity can depend on the beam conditions. For the same total dose delivered, the induced activity can change somewhat if the dose delivery time is different. An example of this is shown in **Figure 4**, where two reconstructed PET images acquired in two different days are displayed. Here the total delivered dose was the same, but the dose delivery and acquisition times were different. These differences cannot be simply corrected by normalizing, because the PET signal is time dependent. Therefore we used only those fractions with similar temporal profiles in the analysis. The number of such fractions is given in the fifth column of **Table 1**.

To reduce salt-and-pepper noise in the images we applied a 5 mm radius median filter. According to the couch angle, the PET images could be rotated into the CT imaging coordinate system expressed in International Electrotechnical Commission (IEC) standard. **Figure 1C** gives an example of a rotated image.

### 2.5 Analysis

The analysis was divided into two parts. First, we wished to investigate the level of experimental fluctuations in inter-fractional range differences observed by the INSIDE system, referred to as sensitivity. This was done by studying inter-fractional range shifts for patients that had no morphological changes (see **Table 1**). Second, we compared these results with patients that did show morphological changes. For the latter patients, we also verified whether the region of the observed range differences is correlated to the presence of real morphological changes in the CT scan.

The strategy that we followed for the analysis is based on an interfractional comparison between a reference PET image and an image acquired during a subsequent fraction *j*. In order to mitigate statistical fluctuations, the reference image was calculated as the average of the first two monitored fractions.

#### 2.5.1 Most-Likely-Shift method

To compare the PET images of the subsequently monitored fractions with the reference image, we used the Most-Likely-Shift method, originally proposed by Frey et al. for off-line PET monitoring images (19) and recently applied also in (33). This is the first time that the MLS method was applied to IB-PET images. Given that such images suffer from artifacts due to the partial angular coverage and limited (see **Figure 1**), it is not a-priori obvious whether the MLS method works.

In summary, given a PET image of a monitored fraction *j*, for each pair of coordinates (*x,y*) in the transverse plane (in the INSIDE reference system), we compared the 1-D activity profile along the beam direction *z* in their distal fall-off with the corresponding reference profile.The MLS method considers profiles belonging to two different treatment fractions for the same pair of coordinates in the transverse plane (*x,y*) and calculates the most probable shift necessary to align these two profiles, normalised at their maximum, along the beam direction. We included only those (*x,y*) pairs into the analysis that were part of the expected activity mask. Moreover, to exclude profiles with low activity from the analysis, we included here only those profiles with an integrated activity of at least 30% of the profile with highest integrated activity. The reader is referred to the original paper by Frey et al. (19) for a detailed description of the MLS method.

We applied the method exactly as described in Frey et al., with three exceptions. First, the value for *z_min_* (*x,y*) (the *z*-value of that activity profile where to start the analysis) was set at 15% of the maximum of the normalized reference profile at coordinates *x,y*. Second, *z_max_* (*x,y*), i.e., the *z*-value where to end the analysis, was set at 2% of the maximum of the normalized reference profile. These modifications were done in order to focus more on the distal fall-off part of the profiles. Third, the shift value δ was limited between -16 and 16 mm, with steps of 1 mm. An algorithm was implemented that provided for each *x*,*y* pair the optimal shift distance *δ_MLS_*. The calculation of the range difference *δR_MLS_* is done by minimizing the absolute profile differences in the distal part (*z_MLS_* ≤ *z* ≤ *z_max_*) of two activity depth profiles, shifted against each other, as follows (19, 34):


(1) $$\delta R_{MLS}=\arg\;\underset{\delta_{MLS}}{\min}\left(\sum_{z_{MLS}}^{z_{\max}}\left|A_{ref}\left(z\right)-A_{j}\left(z-\delta_{MLS}\right)\right|\right)$$

with *A_ref_* and *A_j_* corresponding to the reference activity profile and that of the fraction to be compared, respectively.

Then, a distribution of all the *δR_MLS_* (*x*,*y*)values was created for each fraction *j*. These distributions were fitted with an asymmetric skew-normal distribution and its mean, *μ_MLS_* , and standard deviation, *σ_MLS_* , were evaluated for all monitored fractions *j* of a given patient. For each patient, we then evaluated:

- the average of *μ_MLS_* over all the fractions. This average is denoted by *Δ_MLS_*, giving an indication of the size of typical inter-fractional range shifts between the reference image and the subsequent fractions for this patient;
- the average of *σ_MLS_* over all the fractions, indicated by *∑_MLS_*, giving an indication of the fluctuations in such shifts for a given patient.

Then, to investigate the level of fluctuations in our PET data in absence of any morphological changes, we extracted for the six patients without any morphological changes the average of *∑_MLS_* over these six patients, yielding〈*σ_MLS_* 〉. Such inter-fractional range fluctuations are accidental and can be due to the low statistics of the images, patient setup-errors, INSIDE cart setup errors, small differences in irradiation conditions, and so on.

Then, for each patient and each analyzed fraction, a three-dimensional outliers map *O_MLS_* (*x*,*y*,*z*) of anomalously large shifts was created in order to visualise the largest range difference obtained. The map *O_MLS_* (*x*,*y*,*z*) was filled for each voxel with coordinate *x*,*y*,*z*, which had *z* ≤ *z_min_* as:


(2) $$\{\begin{array}{l} O_{\text{MLS}}\left(x,y,z\right)=\delta R_{\text{MLS}}\left(x,y\right),\text{if }\delta R_{\text{MLS}}\left(x,y\right)\leq -2\cdot \langle \sigma_{MLS}\rangle \text{or if }\delta R_{\text{MLS}}\left(x,y\right)\geq +2\cdot \langle \sigma_{MLS}\rangle \\ O_{\text{MLS}}\left(x,y,z\right)=0\text{ otherwise} \end{array}$$

For *z* > *z_min_* (close to the end of range), *O*_MLS_(*x*,*y*,*z*) = 0. Thus, the outliers maps are represented as 3-dimensional distributions, but each point along the *z*-axis was filled by copying *δR_MLS_* (*x*,*y*) from z=0 up to *z* = *z_min_*. These maps graphically identify the position of the distal fall-off part of the activity distribution, and they highlight the entire region along a pencil beam path that is possibly affected by a range shift with respect to the reference. These maps were re-oriented on the patient’s CT refersence frame, in order to verify if the real morphological change seen in the CT was identified.

The implementation was validated as in (19), i.e., by creating artificially shifted PET distributions and verifying that the MLS analysis correctly identified the introduced shift.

#### 2.5.2 Comparison with Beam’s Eye View method

To confirm the validity of the Most-Likely-Shift method for our PET images, we compared the result with another existing range verification method that performs interfractional comparisons based on 1-D activity profiles: the Beam Eye View method. Details can be found elsewhere (20, 22, 35). This method is based on a multi-threshold approach to extract iso-activity surfaces. Thresholds from 2% up to 8% on the maximum of the entire PET image at 0.5% steps were considered. Briefly summarizing, similar to the MLS method, for each pair of coordinates *x*,*y* in the transverse plane (in the INSIDE reference system), the profile belonging to treatment fraction *j* was compared with the profile of the reference image *A_ref_*. For a given threshold *t*, the shift between these two profiles, *δR*_BEV,t_(*x*,*y*), was defined to be the difference between the maximum depth value reached at threshold for the reference profile, $R_{t}^{ref}\left(x,y\right)$ and that for a monitored fraction, $R_{t}^{j}\left(x,y\right)$ . To obtain the final value of the range difference between the profiles at a given *x*,*y* point, *δR*_BEV_(*x*,*y*), the average was taken over the thresholds. In other words:


(3) $$\delta R_{\text{BEV}}\left(x,y\right)=\frac{1}{N}\sum_{t=1}^{N}\delta R_{\text{BEV},\text{t}}\left(x,y\right)=\frac{1}{N}\sum_{t=1}^{N}\left(R_{t}^{ref}\left(x,y\right)-R_{t}^{j}\left(x,y\right)\right)$$

where *N* is the total number of thresholds considered, which was 13 in our case.

The remaining procedure is exactly as done for the MLS method. For each patient a distribution, containing all the *δR*_BEV_(*x*,*y*) value, was created for each fraction *j*. These were fitted with an asymmetric skew-normal distribution, yielding the mean *μ_BEV_* and standard deviation *σ_BEV_* , for each monitored fraction *j*. For each patient we then extracted:

- the average of *μ_BEV_* over all the fractions, denoted by Δ*_BEV_*.
- the average of *σ_BEV_* over all the fractions, indicated by ∑*_BEV_*.

We extracted for the six patients without any morphological changes the average of ∑*_BEV_*, yielding <*σ_BEV_* > .

For each patient and each analyzed fraction, a three-dimensional outliers map *O*_BEV_ (x, y, z) was created, whose interpretation is the same as for the MLS maps. For each voxel (*x*,*y*,*z*), the map was filled when $z\leq R_{t}^{j}\left(x,y\right)$ , with *t*=8%, as:


(4) $$\{\begin{array}{ll} O_{\text{BEV}}\left(x,y,z\right)=\delta R_{\text{BEV}}\left(x,y\right) & \text{if}\;\delta R_{\text{BEV}}\left(x,y\right)\leq -2\cdot {\mathrm{<\sigma}}_{BEV}>\text{ or if }\delta R_{\text{BEV}}\left(x,y\right)\geq +2\cdot <\sigma_{BEV}>\\ O_{\text{BEV}}\left(x,y,z\right)=0 & \text{otherwise} \end{array}$$

For $z>R_{t}^{j}\left(x,y\right)$ , with *t*=8%, we put *O_BEV_* (*x*,*y*,*z*) = 0. Then the *O_BEV_* (*x*,*y*,*z*) maps were superimposed to the CT images, so that they could be compared with the *O_MLS_* (*x*,*y*,*z*) maps as well as with the CT.

The BEV implementation was validated by creating artificially shifted distributions, and verifying that the analysis correctly identified the shift.

## 3 Results

**Figure 5:**
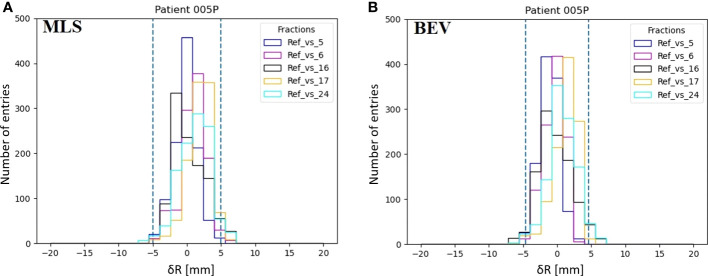
Examples of the distributions of inter-fractional range differences *δR*(*x*,*y*) of various fractions with respect to the reference (M) in patient 005P for the MLS method **(A)** and the BEV method **(B)**. The dashed lines represent the 95% confidence interval tailored for, the MLS (5.0 mm) and the BEV method (4.6 mm) as 2<σ>.

In **Figure 5A** we show an example of a distribution of range differences *δR_MLS_* with , respect to the reference for a few of the available fractions in a patient that does not change (005P). Each histogram entry represents a value of *δR*(*x*,*y*). The same is given for the BEV method in **Figure 5B**.

**Figure 6:**
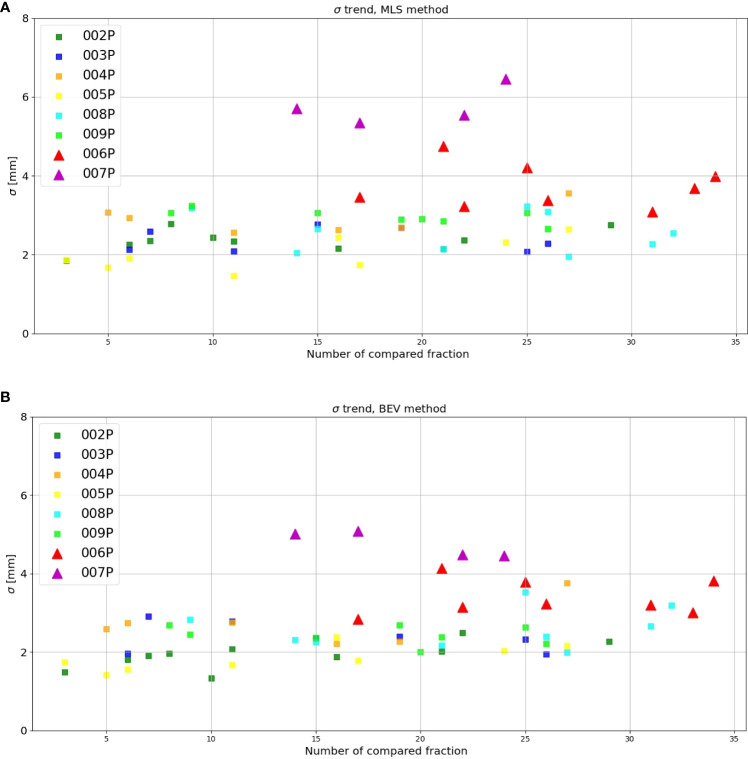
Inter-fractional standard deviation of the range difference distribution as obtained with the **(A)** MLS and **(B)** BEV method for each patient (y-axis) as a function of the number of the fraction compared with the reference (x-axis). The triangles stand for those patients where morphological changes where identified in the control CT, while the squares represent the patients that did not show changes.

**Figure 6A** gives the interfractional standard deviation *σ_MLS_* for each patient with MLS method as a function of the fraction compared with the reference. Squares and triangles stand for the standard deviation obtained for the set of unchanged and changed patients, respectively. For the patients that do not change (squares), we observe that the values for *σ_MLS_* are somewhat lower than 3 mm, the spatial resolution of the INSIDE PET scanner (16). Moreover, we see for these patients that the standard deviation is roughly constant during the course of the treatment. For patients subject to morphological changes (triangles), the standard deviation values mostly exceed 3 mm. In particular for patient 007P, we see that after the 14th fractions *σ_MLS_* is larger than 5 mm.

**Figure 6B** is the same as **Figure 6A**, but now for the BEV method. We see that the results are in agreement with **Figure 6A**: the unchanged patients (squares) had generally a lower standard deviation than the changed patients (triangles), the values are mostly below 3 mm, and the values for the patients that changed are larger.

**Table 2** summarizes the results for each patient obtained by the MLS method in terms of average range activity difference observed over all the fractions with respect to the reference. In the first, second and third column, we report the patient ID, Δ*_MLS_* and *∑_MLS_*, respectively. The reported error for Δ*_MLS_* and *∑_MLS_* is the standard deviation calculated over the various fractions, considering all fractions as independent measurements. The average over the fractions for all the patients that did not present anatomy changes, <*σ_MLS_* > (see Section *Most-Likely-Shift method*), was calculated to be 2.5 mm. For the two patients that changed, the values for *∑_MLS_* were found to be larger.

**Table 2:** For each patient, the average inter-fractional range difference Δ*_MLS_* and average standard deviation *∑_MLS_* obtained with the MLS method, together with the corresponding values Δ*_BEV_* and *∑_MLS_* for the BEV method.

Patient	Δ*_MLS_* [mm]	*∑_MLS_* [mm]	Δ*_BEV_*[mm]	*∑_BEV_*[mm]
002P	0.2±1.0	2.3±0.3	0.3±0.9	1.9±0.3
003P	1.2±0.7	2.4±0.3	0.1±1.0	2.4±0.3
004P	-1.0±0.7	2.9±0.3	-0.6±0.9	2.7±0.5
005P	1.0±1.1	2.0±0.4	0.3±1.4	1.8±0.4
008P	2.0±0.8	2.6±0.5	1.5±0.6	2.6±0.5
009P	-0.6±0.9	3.0±0.2	-0.1±0.8	2.4±0.2
006P	1.2±0.6	3.7±0.5	1.6±1.1	3.4±0.4
007P	-0.8±1.1	5.8±0.4	-2.6±1.8	4.8±0.3

The corresponding values for the BEV method, Δ*_BEV_* and *∑_BEV_*, are given in the fourth and fifth column of **Table 2**, respectively. The average over the fractions for all the patients that did not present anatomychanges, <*σ_BEV_* > (see Section *Comparison with Beam’s Eye View method*), was calculated to be 2.3 mm. Again, for the two patients that changed, the values for *∑_BEV_* were found to be larger than those for patients that did not change.

To establish whether the group of changed patients was statistically different from the group of unchanged patients, we performed the Student’s t-test over the values of Δ and *∑*. While the Δ values were not statistically different (for the MLS method *t* = 0.27, *p* = 0.79, and for the BEV method *t* = 0.67, *p* = 0.53), for the *∑* values we found a significant difference (for the MLS method *t* = -3.90, *p* = 0.008, and for the BEV method *t* = -4.19, *p* = 0.006).

**Figure 7:**
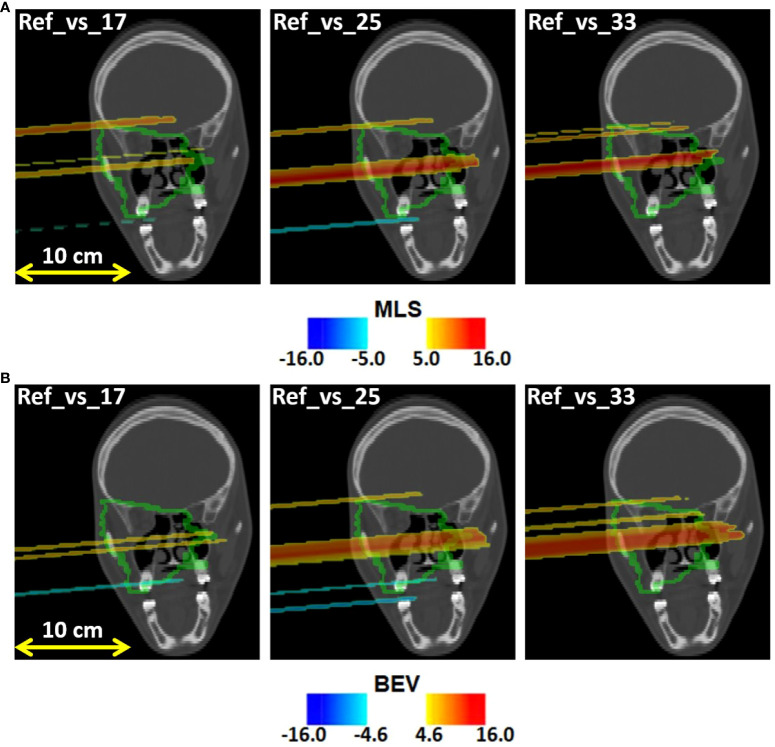
Coronal views of the control CT of patient 006P, with the **(A)** MLS outliers map *O_MLS_* (*x*,*y*,*z*) and the **(B)** BEV outliers map *O_BEV_* (*x*,*y*,*z*) superimposed, obtained by comparing fraction 17, fraction 25, and fraction 33 with the reference. The colored areas are the pencil beam paths that lead to anomalous range differences with respect to the reference, with red indicating a range overshoot with respect to the initial situation. The overshoot is especially pronounced in fraction 25 and 33. The colormaps were obtained as described in 2.5.1 and 2.5.2 for the MLS and the BEV method, respectively.

**Figure 7A** displays the outliers *O_MLS_* (*x*,*y*,*z*) maps obtained for patient 006P by the MLS method. This is a patient which was subject to small morphological changes, as observed by the radiation oncologist and demonstrated in **Figure 2**. The colored area represents the pencil beam paths that had anomalous range differences with respect to the reference, see Eq. 2. The red color indicates a positive range difference with respect to the initial situation (overshoot). We see that the red color becomes darker as the number of the treatment fractions increases, strongly indicating a range overshoot that increases along the treatment course. In the penultimate fraction (fraction 33) of the treatment course, we observe range differences as large as 16 mm, i.e., a substantial range overshoot. The cause of the range overshoot becomes clear when looking at **Figure 2**. Comparing **Figures 2A**–**C** (planning CT) with **Figures 2D**–**F** (control CT), a cavity is seen that is emptied in the control CT. The fact that the regions highlighted in **Figure 7A** cover those of the anatomical change in the control CT is a strong indication that our PET images are sensitive to such changes. We therefore believe that the MLS method can be successfully applied to IB-PET images to detect morphological changes like cavity emptying.

**Figure 7B** shows the outliers maps *O_BEV_* (*x*,*y*,*z*) obtained for patient 006P by the BEV method. The colored areas are seen to be similar to those of **Figure 7A**. Thus, the MLS and BEV method are roughly in agreement.

**Figure 8:**
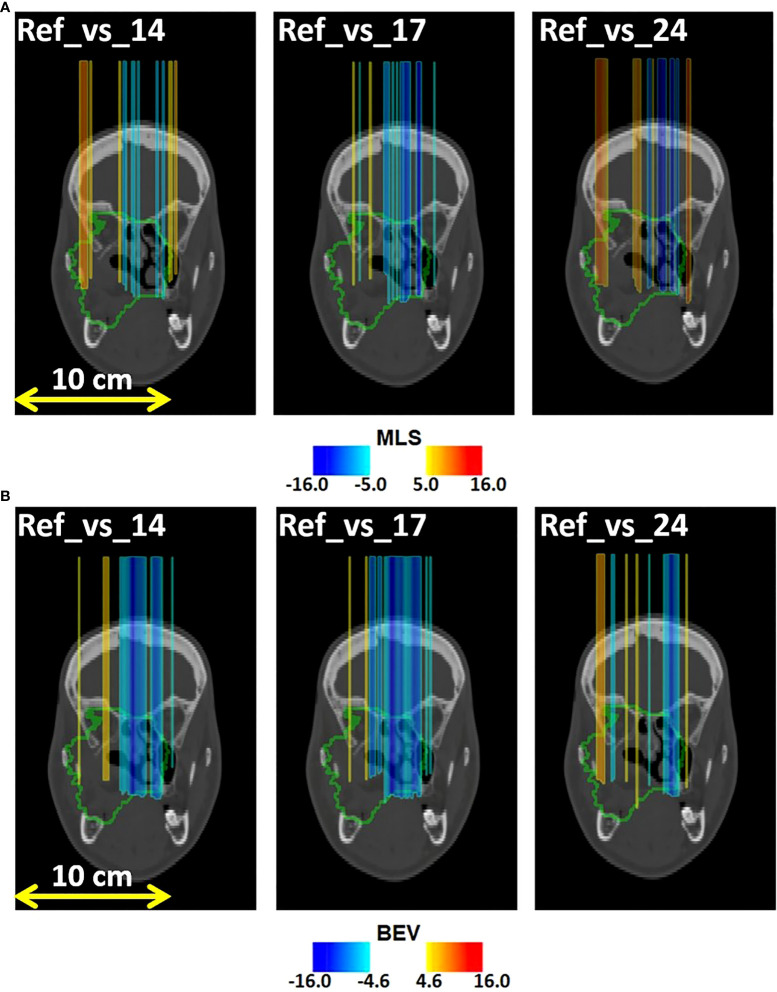
Coronal views of the control CT of patient 007P, with superimposed the **(A)** MLS outliers map *O_MLS_* (*x*,*y*,*z*) and **(B)** BEV outliers map *O_BEV_* (*x*,*y*,*z*) , obtained by comparing fraction 14, fraction 17, and fraction 24 with the reference. The colored areas are the pencil beam paths that lead to anomalous range differences with respect to the reference, with red and blue indicating a range overshoot and undershoot with respect to the initial situation, respectively. Most clear are the beam undershoots (the blue areas). The colormaps were obtained as described in 2.5.1 and 2.5.2 for the MLS and the BEV method, respectively.

In **Figure 8A** we display the outliers maps *O_MLS_* (*x*,*y*,*z*) for patient 007P by the MLS method. In this case, the map shows both range overshoots with respect to the reference (identified by the red color) and undershoots (identified by the blue color). By comparing **Figure 3** with **Figure 8A**, we see that the region where the cavity has emptied is only weakly highlighted (orange). The blue zones that are identified may correspond to inflammation effects (see discussion).

Finally, in **Figure 8B** we demonstrate the outliers maps *O_BEV_* (*x*,*y*,*z*) , obtained for patient 007P by the BEV method. Also in this case the zones that are highlighted are those affected by beam overshoots (identified by the red color) and undershoots (identified by the blue color) with respect to the reference. They are similar to the regions that were identified with the MLS method, but the colored regions are somewhat larger (see discussion).

## 4 Discussion

In this work we analyzed for the first time all of the available IB-PET patient data for proton therapy treatments monitored during the INSIDE clinical trial. To evaluate the experimental level of inter-fractional fluctuations in IB-PET images in absence of anatomical changes (sensitivity), we studied six patients that did not show morphological changes. Using the MLS method we found for these patients an overall standard deviation <*σ_MLS_* > in activity range difference of 2.5 mm. This is smaller than 3 mm, which is the spatial resolution of the INSIDE PET scanner  (16).

Regarding the observed differences between changed and unchanged patients, we saw that the average standard deviations ∑*_MLS_* were larger for changed patients than those for unchanged patients. Moreover, the outliers maps in **Figures 7A**, **8A** include approximately the regions that were also seen to change in the control CT. In patient 007P, the situation is complicated because the beam goes through highly heterogeneous tissue. Still, the results are encouraging, given the small size of the anatomical changes (order of a few ml).

We evaluated to what extend the values in **Table 2** for the MLS analysis were affected by the choice of the parameters chosen (see Section *Most-Likely-Shift method*). Varying the threshold value for inclusion of the profiles from 30% (default) to 20% and 35%, the results did not change significantly. Also, varying the *z_min_* (default 15%) between 10% and 20%, no significant range differences were observed. Regarding *z_max_* (default 2%), this value should be as small as possible in order to include as much activity in the fall-off region as possible. However the noise level was found to be 2% so using lower values resulted in inconsistent results. Thus, the results were sufficiently robust.

Comparing the MLS and BEV methods, we saw that the MLS method gave results that were similar to those obtained with the BEV method for all patients. Thus, the methods are mostly in agreement with each other. An example where the methods gave slightly different results was in patient 007P (see **Figures 8A**, **B**), where the identified zones were partly different. The advantage of the MLS method with respect to the BEV method is that it evaluates point by point the most suitable shift to align two profiles, and does not only look at the most distal values like the BEV method.

We also verified that our results for the BEV method were in agreement with those published previously for three of the eight patients (20). Small differences could be attributed to differences in the fit procedure and in the choice of the reference PET image, which was in the case of (20) defined to be the first acquired PET fraction, while we took the mean of the first two acquired images as reference.

Several aspects in the analysis deserve more discussion. First, the number of analyzed patients and the number of fractions that could be included in the analysis was small (**Table 1**). The differences between changed and unchanged patients could in principle have been caused by other factors, including the small statistics of the PET images, differences in patient setup, tumor type, tumor size, depth of the irradiated region, irradiation, etc. The influence of these sources of uncertainty should be investigated in more detail by monitoring more patients. At the same time, the outliers maps agreed approximately with the zones that were seen to change in the control CT. Thus, we believe that the INSIDE IB-PET images can give indications about inter-fractional range differences. The MLS and BEV methods can both be applied for this purpose. This was especially visible in **Figures 7**, **8**, where the colors indicating beam overshoots and undershoots become more intense as the number of treatment fractions increased. However, the effect is small and more patient data are needed to confirm whether such trends through the course of treatment are observable.

Second, it must be noted that we monitored only one of the fields, i.e., the first field delivered. In some cases we had *N_fields_* = 3 (see **Table 1**), resulting in a very limited overall statistics of the PET signal. This can possibly be improved by assuring that the field delivered first is the one with largest activity, or by combining fields (36).

Finally, the size of the morphological changes of patient 6 and 7 was small (order of a few ml). In fact, these changes were considered small enough to not require replanning, since the recalculation of the treatment plan on such modified anatomy did not show any clinically significant modification of the DVHs both for target and OARs. Increasing the number of patients with larger expected changes is foreseen for the second phase of the INSIDE clinical trial. This can better confirm the validity of the INSIDE in-beam PET system in detecting morphological changes.

Despite the small number of patients and fractions that could be included in this study, the above results are encouraging. For future in-beam PET data acquisitions, we suggest to only compare PET images which are acquired under approximately the same irradiation conditions, to monitor the first field delivered to avoid contamination from other fields, and to monitor as many fractions as possible.

## 5 Conclusion

In this work we performed an inter-fractional range difference analysis including all the available patient data for proton treatments acquired during the INSIDE clinical trial: six patients not subject to morphological change, and two patients subject to morphological change. We applied the Most-Likely-Shift (MLS) method for detecting inter-fractional range differences, which, to the best of our knowledge, was the first time it was applied for IB-PET images. It was compared with the Beam Eye View (BEV), that was previously applied to a subset of the patients included in the clinical trial.

When putting together all fractions and all patients that did not have morphological changes, the standard deviation of the range variations in activity profiles, <*σ_MLS_* >, was found to be 2.5 mm with the MLS method. The corresponding value for the BEV method was <*σ_BEV_* > =2.3 mm. On the other hand, for patients where anatomical changes occurred, we found larger standard deviation values.

For the patients with anatomical changes we created outliers maps to indicate anomalous range variations. These were superimposed on the control CT, and the regions affected by an anomalous range difference approximately covered those with real anatomical variations observed in the control CT. The results are encouraging and suggest that the MLS method can possibly be used as a support tool by clinical personnel to detect anomalous situations during the treatment course and to guarantee the effectiveness of the treatment plan.

## Data availability statement

The data that support the findings of this study are partly available on request from the corresponding author. The data are not publicly available due to privacy or ethical restrictions.

## Ethics statement

The studies involving human participants were reviewed and approved by Comitato etico del Policlinico San Matteo di Pavia. The patients/participants provided their written informed consent to participate in this study.

## Author contributions

The analysis and manuscript was prepared by MMog, ACK and MGB. All authors have contributed to the publication, being variously involved in the design and the construction of the detectors, in writing software, calibrating subsystems, operating the detectors, acquiring data and finally analysing the processed data. The INSIDE Collaboration members discussed and approved the scientific results reported in the submitted document. The work was subject to an internal collaboration-wide review process.

## Funding

The INSIDE project was funded by the Italian Ministry of Education (PRIN MIUR 2010P98A75, 2013–2016), the Italian Institute of Nuclear Physics (RDH and INFN-RT2 PETRA 172800 projects, since 2016), the Historical Museum of Physics and the Enrico Fermi Study and Research Center, the Tuscany Government (POR FSE 2014–2020, INFN-RT2 PETRA 172800 Project, 2018-2019), and the CNAO Foundation (INSIDE2, since 2017).

## Conflict of interest

The authors declare that the research was conducted in the absence of any commercial or financial relationships that could be construed as a potential conflict of interest. All authors contributed to the article and approved the submitted version.

## Publisher’s note

All claims expressed in this article are solely those of the authors and do not necessarily represent those of their affiliated organizations, or those of the publisher, the editors and the reviewers. Any product that may be evaluated in this article, or claim that may be made by its manufacturer, is not guaranteed or endorsed by the publisher.
